# Identifying genetic variants underlying medication-induced osteonecrosis of the jaw in cancer and osteoporosis: a case control study

**DOI:** 10.1186/s12967-019-2129-3

**Published:** 2019-11-20

**Authors:** Kye Hwa Lee, Su-Hwan Kim, Chang Hyen Kim, Byung Joo Min, Grace Juyun Kim, Younggyun Lim, Hun-Sung Kim, Kang-Min Ahn, Ju Han Kim

**Affiliations:** 1 Center for Precision Medicine, Seoul National University Hospital, Seoul, 03082 South Korea; 2 Department of Periodontics, Asan Medical Center, Seoul, 05505 South Korea; 3 Department of Dentistry, University of Ulsan College of Medicine, Seoul, 05505 South Korea; 4 Department of Oral and Maxillofacial Surgery, Seoul St. Mary’s Hospital, College of Medicine, The Catholic University of Korea, Seoul, 06591 South Korea; 5 Division of Biomedical Informatics, Seoul National University Biomedical Informatics (SNUBI) and Systems Biomedical Informatics Research Center, Seoul National University College of Medicine, Seoul, 03080 South Korea; 6 Department of Medical Informatics, College of Medicine, The Catholic University of Korea, Seoul, 06591 Republic of Korea; 7 Department of Oral and Maxillofacial Surgery, University of Ulsan College of Medicine, Seoul, 05505 South Korea

**Keywords:** Osteoporosis, Angiogenesis, Pharmacogenomics, Bioinformatics, Cancer biology

## Abstract

**Background:**

Bisphosphonate-induced osteonecrosis of the jaw (BRONJ) presents with a typical pattern of jaw necrosis in patients who have been prescribed bisphosphonates (BPs) and other antiangiogenetic drugs to treat osteoporosis or bone-related complications of cancer.

**Methods:**

This study divided 38 patients with BRONJ into two groups according to the prescribing causes: cancer (*n *= 13) and osteoporosis (*n *= 25), and underwent whole exome sequencing and compared them with normal controls (*n *= 90). To identify candidate genes and variants, we conducted three analyses: a traditional genetic model, gene-wise variant score burden, and rare-variant analysis methods.

**Results:**

The stop-gain mutation (rs117889746) of the *PZP* gene in the BRONJ cancer group was significantly identified in the additive trend model analysis. In the cancer group, *ARIDS*, *HEBP1*, *LTBP1*, and *PLVAP* were identified as candidate genes. In the osteoporosis group, *VEGFA*, *DFFA*, and *FAM193A* genes showed a significant association. No significant genes were identified in the rare-variant analysis pipeline. Biologically accountable functions related to BRONJ occurrence-angiogenesis-related signaling (*VEGFA* and *PLVAP* genes), TGF-β signaling (*LTBP1* and *PZP* genes), heme toxicity (*HEBP1*) and osteoblast maturation (*ARIDS*)-were shown in candidate genes.

**Conclusion:**

This study showed that the candidate causative genes contributing to the development of BRONJ differ according to the BP dose and background disease.

## Background

Bisphosphonates (BPs) are antiresorptive agents that are commonly used to treat osteoporosis, multiple myeloma, and metastatic solid tumors [1]. BPs become internalized into osteoclasts via endocytosis and subsequently inhibit their activity [2]. Despite BPs being well known to be effective in preventing osteoporotic fractures and preventing cancer-related side effects in bone, there has been a sharp decline in BP prescriptions in recent years, from 21.3 million in 2002 to 14.7 million in 2012 in the United States, with increasing reports of diverse rare but serious side effects associated with the use of BPs [3]. Osteonecrosis of the jaw (ONJ) is one of the most-serious and well-known side effects of BPs [4]. ONJ is characterized by jawbone necrosis, which exposes necrotic bones through holes in mucous membranes or the facial skin, traditionally ranging from a few millimeters to a few centimeters [2, 5]. The reported incidence rate of ONJ when using BPs to treat cancer has ranged from 0.7 to 10.3% [6], while its incidence in osteoporosis has not been established [5, 7]. Because other drugs are also known to be risk factors for ONJ, such as other types of antiresorptive drug (denosumab) and antiangiogenics, the American Association of Oral and Maxillofacial Surgeons committee recommended to change the nomenclature of ONJ caused by drugs from BRONJ (bisphosphonate-related osteonecrosis of the jaw) to medication-related osteonecrosis of the jaw (MRONJ) in 2014 [8].

Apart from the well-known direct causes of MRONJ such as dental surgery or gingival infection, the main mechanism underlying the occurrence of MRONJ has not been clearly elucidated [9]. Since ONJ occurs only in specific individuals, multiple studies have been carried out to confirm the genetic background of MRONJ [9–11]. Despite the dearth of genomic studies and their results not being well replicated, these studies have added a deep pathological understanding of and insight into the development of MRONJ, such as the patient’s innate immunity, angiogenesis inhibition, osteoclast suppression, and systemic/local inflammations being strong predisposing factors [8]. Some of the candidate genes identified by these studies are *TGFb1*, *MMP2*, *PPARG*, *CYP2C8*, *VEGF*, *COL1A1*, *RANK*, *OPG*, *OPN*, and *RBMS3* [12–16]. However, most of these studies were either candidate-gene studies or genome-wide association studies (GWASs) [17]. Previous whole exome sequencing (WES) studies have found that multiple biological pathway contribute to the occurrence of MRONJ, but no specific contributing genes have been identified [9]. Recently, a study included total 44 multiple myeloma and 17 solid tumor BRONJ patients of European ancestry using WES was identified protective SNPs with significant linkage disequilibrium with *SIRT1* and *HERC4* genes [15, 18].

In this study we applied case–control methods that are commonly used in genomics research on complex diseases to identify genes exhibiting large variations between BRONJ patients and healthy control subjects. We divided BRONJ patients into two groups depending on whether BPs had been prescribed for cancer and osteoporosis, based on the assumption that the genetic vulnerabilities contributing to the occurrence of BRONJ differ between the long-term accumulation of BPs in osteoporosis and the high-dose toxicity of BPs in cancer.

## Methods

### Study design and participants

We prospectively collected clinical data and blood and saliva samples from 40 patients diagnosed with BRONJ: 30 at Asan Medical Center from May 2013 to November 2015 and 10 at Seoul St. Mary’s Hospital from November 2010 to November 2014. All of the patients were clinically evaluated by dentists and were diagnosed as BRONJ according to the guideline from the American Association of Oral and Maxillofacial Surgeons [8]. All patients had been taken BPs or had a history of BPs prescription before ONJ occurs. They had necrotic lesions in maxillar or mandibular bone and no history of administration of radiation therapy at the necrotic bone area. We performed sample size estimation with 70% detection power, 20% significance level, MAF in case and MAF in 1–5% control, respectively, to identify variations contributing to BRONJ. From the calculation, 16–39 and 48–116 samples were required for the case and control respectively. Therefore, in this study, we started the study with 40 cases and 90 health controls. Excluding two patients who were failed DNA extraction, 38 patients were included in the final study. BPs were prescribed for cancer (multiple myeloma or metastatic cancer) in 13 patients and for osteoporosis in 25 patients. The types of BPs taken by the patients were zoledronate (*n *= 11), alendronate (*n *= 10), risedronate (*n *= 5), pamidronate (*n *= 3), ibandronate (*n *= 1), zoledronate/ibandronate (*n *= 1), or unknown (*n *= 6). The most commonly used drug was zoledronate (61%, *n *= 8) in the cancer patients and alendronate (40%, *n *= 10) in the osteoporosis patients. Before the onset of BRONJ, BPs had been prescribed for 18.1 ± 13.9 months (mean ± SD) in the cancer patients and 56.0 ± 52.3 months in the osteoporosis patients. The clinical phenotypes of the patients are listed in Table 1. The control subjects were enrolled for the previous study of “Physicians’ Seq Project” [19]. These normal control group was from the previous sequencing study of physicians to evaluate the physicians’ expectation and attitude for pharmacogenomics using their own genome. A detailed description was in the previous research paper. The incidence of BRONJ among BP recipients was reported as one in 100,000, which is similar to the occurrence of jaw osteonecrosis (ONJ) in the general population [7, 20, 21]. Thus, we considered that those who had osteoporosis but did not develop ONJ within 6 months to a year would not have significantly different genetic characteristics than the general population.

**Table 1:** Clinical characteristics of the study participants

	BRONJ total cases (*n *= 38)	BRONJ cancer cases (*n *= 13)	BRONJ osteoporosis cases (*n *= 25)	Normal controls (*n *= 90)
Age, years	79.9 ± 11.7	60.1 ± 8.7	76.6 ± 8.8	41.1 ± 7.2
Males	8 (21%)	7 (53.8%)	2 (8.0%)	47 (52.2%)
Diagnosed condition resulting in BP prescription				–
Cancer	13 (34.2%)	13 (100%)	0 (0%)	
Breast cancer	6 (46.2%)	6 (46.2%)	–	
Multiple myeloma	5 (38.5%)	5 (38.5%)	–	
Prostate cancer	2 (15.4%)	2 (15.4%)	–	
Osteoporosis	25 (65.7%)	–	25 (100%)	
BP				–
Zoledronate	11 (29.0%)	8 (61.5%)	3 (12.0%)	
Alendronate	10 (26.0%)	–	10 (40.0%)	
Residronate	5 (13.2%)	–	5 (20.0%)	
Pamidronate	3 (7.9%)	3 (23.1%)	–	
Ibandronate	2 (5.3%)	–	2 (8.0%)	
Zoledronate/ibandronate	1 (2.6%)	1 (7.7%)	–	
Unknown	6 (15.8%)	1 (7.7%)	5 (20.0%)	
Duration of BP treatment before BRONJ occurrence, months	42.7 ± 46.3	18.1 ± 13.9	56.0 ± 52.3	–

Data are mean ± SD or *n* (%) values

### Sequencing data analysis

The detailed methods of WES, variant calls, quality control and annotation were described in Additional file 1: Materials and Methods. Because the case group comprised subjects with two causes of disease for BP prescriptions that also showed significantly different clinical features, we divided the cases into two subgroups: the BRONJ cancer group (BC, *n *= 13) and the BRONJ osteoporosis group (BO, *n *= 25) as shown in Additional file 1: Figure S1.

**Fig. 1:**
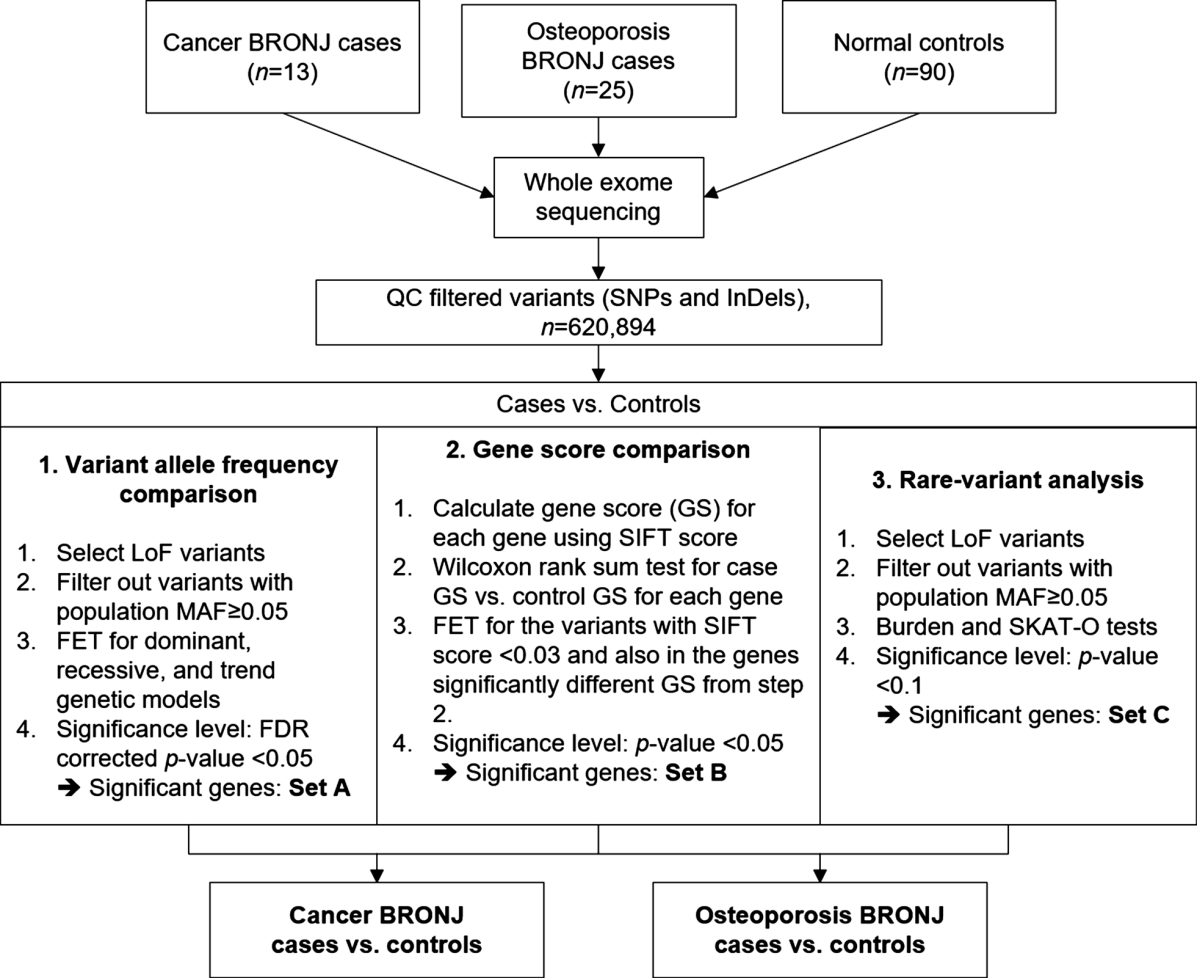
The three analysis pipelines for identifying candidate genes and variants

Because BRONJ patients in our study were mixed with high-dose, intravenous bisphosphonate-treated cancer group and low-dose, per-oral bisphosphonate-treated osteoporosis group, we assumed that the underlying mechanism of ONJ occurrence differ between the two groups, so divided patients into two groups according their background disease and BPs dosage and compared each group with the normal control (*n* = 90). To identify candidate genes and variants associated with BRONJ for each group, we used three analytical methods. Significant genes/variants identified from the three methods were classified using gene set enrichment analysis and we reviewed literatures to evaluate fundamental pathophysiology of BRONJ. The three analysis pipeline is demonstrated in Fig. 1.

We applied three analysis methods to identify BRONJ-associated variants and genes. First, three different traditional genetic testing models were used to compare variant frequencies between the cases and controls. This method has a solid statistical basis but also a low statistical power in detecting variants exhibiting mild effects and low frequencies. Second, collapsing analysis with the gene as a unit of measurement was used to assess the damaging effects of all deleterious variants in the genes. This is similar to other burden-based tests that collapse all rare and/or damaging variants within a region into a single value, but our method yielded a score from 0 to 1 that reflected the variability of the variant distribution in a gene. The third analysis method was the rare-variant association test using a multiple regression model while adjusting covariates in order to identify rare variants that affect the phenotype.

### Case–control test of variant frequencies

We performed a case–control association analysis with the variant-allele frequencies (AFs) using the SnpSift CaseControl tool for three different genetic models [22] for each BC and each BO versus controls. The statistical tests used were the Cochran–Armitage test for trends and Fisher’s exact test (FET) for the dominant and recessive models. For the trend model, we applied weights of 0, 1, and 2 for the reference homozygous, alternative heterozygous, and alternative homozygous variants, respectively. To identify SNPs and INDELs that significantly impacted the function of the corresponding protein, the loss of function (LoF) variants defined as follows were used for further analysis: stop gain/loss, coding INDELs, splice-site acceptors, and splice-site donors. We also included variants predicted as damaging according to their SIFT [23] score and a CADD [24] score of > 20.

### Gene-based collapsing analysis

In order to measure the degree of damage of a specific gene, we used a previous algorithm to convert the number of a mutations in a gene into a gene score [25, 26]. The gene score (a gene deleteriousness score) quantified the impact of damage of a gene, and was defined as the geometric mean of the SIFT scores for the multitude of deleterious variants in a gene. The gene score represents an estimate of the aggregate impact of all deleterious variants in the genes. Since the SIFT score ranges from 0 to 1 and is lower for deleterious variants, a lower gene score indicates greater damage to the function of the gene at the protein level. We identified genes with different gene scores between cases and controls using Wilcoxon rank-sum test. For further analyzing which variants would be responsible for the deleteriousness of a specific gene, we used FET to compare the variant frequencies with all SIFT-mapped variants.

### Rare-variant association analysis

To evaluate the effect of rare functional variants that were only observed as phenotype manifestations in one or two patients, we applied the sequence kernel association test (SKAT) for rare LoF variants [27]. The threshold for rare variants was a MAF < 0.5% in the Asian population of phase I of the 1000 Genomes [28] and the ExAC project [29] from the SnpSift annotation. Functionally damaging variants included previously defined LoF variants. Sex and age were used as covariates, and the threshold for significance was a *p* value of 0.1 after correcting for multiple-tests bias. We used the freely available R package to apply the SKAT with a small-sample option. Statistical analyses including multiple-tests correction were implemented using custom scripts in R (v3.1.5, R Foundation for Statistical Computing, Vienna, Austria) [30].

## Results

### Variant frequency analysis

To identify genetic variants associated with BRONJ, we performed statistical tests with three genetic models—dominant, recessive, and Cochran–Armitage trend models—for the two case groups (13 BC and 25 BO) versus 90 healthy controls. Among 519,375 SNPs/INDELs and 23,420 genes, we extracted 2646 SNPs/INDELs and 2327 genes with LoF variants and performed statistical tests for the BC. LoF variants include stop gain/loss, coding INDELs, splice-site acceptors, splice-site donors, and also variants that were predicted as damaging according to both the SIFT [23] and CADD [24] scores. For BO there were 3684 SNPs/INDELs and 3101 genes with LoF variants out of the total of 693,497 SNPs/INDELs and 23,534 genes. To exclude population-wise major variants that were likely to be benign, variants with a MAF > 0.5% in the Asian population from phase I of the 1000 Genomes Project [28] and the ExAC [29] were filtered out. This left 343 variants in 335 genes for BC and 367 variants in 357 genes for the BO. Using SnpSift case–control analysis, we performed tests for the three genetics models for all of these LoF variants: dominant, recessive, and additive trend models. After correcting for multiple-tests bias, a stop-gain variant in *PZP* for the BC versus controls was identified in the trend model that was associated with a higher BRONJ risk (Table 2). There was no significant variant in BO versus controls.

**Table 2:** Results of the variant-allele-frequency association analysis in BC

Chr	POS	rsID	Ref/Alt	Gene	Function	SIFT/CADD	Genotype counts		Allele frequency		*p value*
							Case (*n *= 13)	Control (*n *= 90)	1KP ASN	ExAC ASN	
							GG/GA/AA	GG/GA/AA			
chr12	9,333,626	rs117889746	G/A	*PZP*^a^	Stop gain	0.3/36	11/1/1	70/20/0	0.095	0.083	0.001

^a^PZP, PZP, alpha-2-macroglobulin like

### Gene-score comparisons

After excluding probably benign hypervariable genes including transcription factors (Additional file 1: Results), we performed a clustering analysis using the DAVID Functional Annotation Clustering Tool [31] to evaluate common biological functions shared in these gene sets on 232 and 564 genes for the BC and BO, respectively. For functional terms mapped to the pathway databases, there were eight clusters for BC versus controls and seven for BO versus controls with a significance of *p *< 0.05. The enrichment score of each group and statistical test results for the BC and BO are listed in Additional file 1: Tables S1, S2, respectively. All of the annotated genes are presented in Additional file 1: Table S3.

**Fig. 2:**
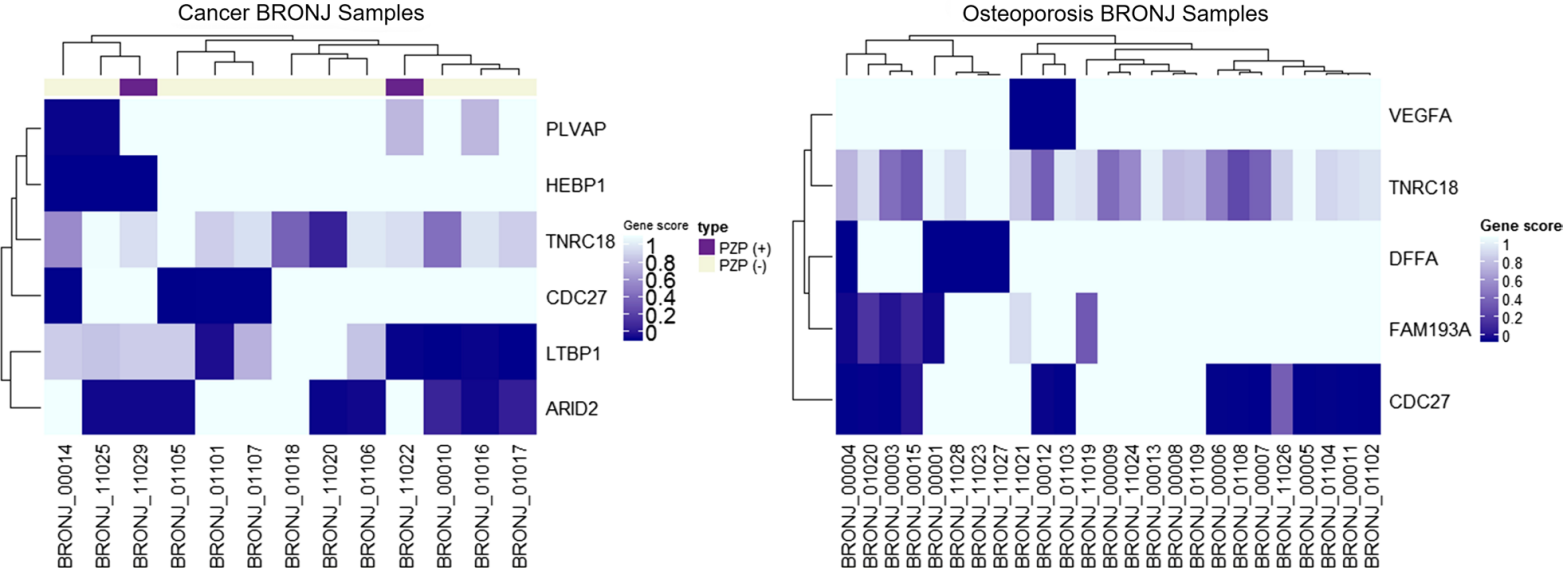
Heat maps showing damaged genes among the BRONJ samples

To identify contributing variants for cases with lower gene scores, we performed a FET of the AF of all variants with SIFT scores of < 0.3 among the functionally damaging variants in the case versus control groups. Functionally damaging variants are defined in Additional file 1: Figure S2. At this step there were 10,087 and 12,952 variants for BC and BO, respectively, among which the number of intersection of variants having significantly different gene scores were 878 and 1647, respectively. Then 161 variants in the BC and 444 variants in the BO with higher AF for cases than controls (*p *< 0.05, odds ratio > 1) were filtered in. To filter out population major variants and false-positive results, we excluded variants with lower AFs in 90 healthy controls than in the ExAC Asian or the 1000 Genomes Project Asian population (12 variants in the BC and 15 variants in the BO). After removing the variants with a zero allele count in the control group, six and five variants were identified and validated using IGV viewer for the BC and BO, respectively. From the gene-score analysis pipeline, we identified *ARID2*, *CDC27*, *HEBP1*, *LTBP1*, *PLVAP*, and *TNRC18* in the BC, and *CDC27*, *DFFA*, *FAM193A*, *TNRC18*, and *VEGFA* in the BO (Tables 3, 4). To identify the clusters of patients for these genes, we drew the heat map for the gene score (Fig. 2).

**Table 3:** Genes and variants identified from the gene-score analysis step for cancer BRONJ cases

No.	Genes					Variants					
	Hugo	Gene name	Gene score		*p value*	Chr	Pos	Ref/Alt	SIFT/CADD	AF	*p value*
			Case	Control						Case/control	
1	ARID2	AT-rich interaction domain 2	0.41 ± 0.49	0.83 ± 0.83	< 0.001	chr12	46,246,206	G/T	0.02/15.55	0.23/0.07	0.015
2	CDC27	Cell division cycle 27	0.69 ± 0.48	0.97 ± 0.18	< 0.001	chr17	45,214,654	C/T	0/20.6	0.15/0.01	0.003
3	HEBP1	Heme-binding protein 1	0.77 ± 0.44	0.98 ± 0.15	0.001	chr12	13,128,263	C/G	0/20.1	0.12/0.01	0.015
4	LTBP1	Latent transforming growth factor beta-binding protein 1	0.52 ± 0.43	0.81 ± 0.24	0.018	chr2	33,505,139	C/A	0/32	0.15/0.03	0.016
5	PLVAP	Plasmalemma vesicle-associated protein	0.81 ± 0.37	0.96 ± 0.16	0.029	chr19	17,476,328	G/A	0.01/6.50	0.08/0.01	0.043
6	TNRC18	Trinucleotide repeat containing 18	0.73 ± 0.27	0.89 ± 0.22	0.003	chr7	5,427,467	G/A	0.01/16.78	0.08/0.01	0.043

*AF* allele frequency

**Table 4:** Genes and variants identified from the gene-score analysis step for osteoporosis BRONJ cases

No.	Genes					Variants					
	Hugo	Gene name	Gene score		*p value*	Chr	Pos	Ref/Alt	SIFT/CADD	AF	*p value*
			Case	Control						Case/Control	
1	CDC27	Cell division cycle 27	0.46 ± 0.49	0.97 ± 0.18	< 0.001	chr17	45,214,654	C/T	0/20.6	0.22/0.01	0.000
2	DFFA	DNA fragmentation factor subunit alpha	0.80 ± 0.41	0.94 ± 0.23	0.022	chr1	10,521,569	C/T	0/7.665	0.10/0.03	0.042
3	FAM193A	Family with sequence similarity 193 member A	0.79 ± 0.38	0.95 ± 0.18	0.009	chr4	2,632,766	C/T	0.02/12.71	0.08/0.01	0.008
4	TNRC18	Trinucleotide repeat containing 18	0.73 ± 0.24	0.89 ± 0.22	< 0.001	chr7	5,427,467	G/A	0.01/16.78	0.08/0.01	0.008
5	VEGFA	Vascular endothelial growth factor A	0.88 ± 0.33	0.98 ± 0.13	0.033	chr6	43,752,346	C/A	0/14.64	0.06/0.01	0.033

*AF* allele frequency

Hierarchical clustering analyses revealed the exclusive pattern of genes related to angiogenesis (*PLVAP*) and genes related to TGF-β signaling (*LTBP1* and *PZP*) in the BC. Despite *TNRC18* and *CDC27* showed significant differences in both BC and BO but those genes do not have definite explainable functions underlying BRONJ. In BO, *DFFA* and *FAM193A* genes also significantly enriched but do not have any known underlying function related to ONJ. Otherwise, *VEFGA* gene in BO and *PLVAP*, *HEBP1*, and *LTBP1* gene in BC could be applied to explain the BRONJ mechanism. *ARID2* gene showed damaged score in eight patients, and also known as cancer-related gene, but hardly having a pathophysiological relationship to BRONJ.

### Rare-variant association analysis

We performed the SKAT-O and burden test for BC versus controls and for BO versus controls including LoF variants with a MAF < 0.05% in the Asian population of the ExAC project and the 1000 Genomes Project phase-I data. The total number of genes with rare LoF variants was 112 in the BC and 121 in the BO. There were no genes consistent with the FDR-corrected probability criterion of *p *< 0.05 in either the BC or BO. For BO versus controls, 60 genes were significantly associated with the risk of BRONJ in the SKAT-O analysis without multiple testing correction, and there were none in the burden test. The biological process GO term for proteolysis (GO:0006508) was significantly enriched (*p *= 0.02), with an enrichment score of 1.21, including six genes (*ENDOU*, *MMP8*, *CPNE1*, *TMPRSS7*, *KLK10*, and *PRSS42*) from the gene set enrichment analysis. There were no genes associated an increased or decreased risk of BRONJ occurrence in the BC.

## Discussion

This study evaluated the genes associated with the predisposition to develop BRONJ by comparing patients according to the reason for them being prescribed BPs (cancer or osteoporosis) using WES. The genes identified in our study—*LTBP1*, *PZP*, *ARID2*, and *HEBP1* in osteoporosis BRON patients—clearly support the previous evidence that angiogenesis, osteoclast activity, bone remodeling, and immune responses are critical underlying mechanisms. In osteoporosis BRONJ group, we identified the *VEGFA* gene which is known to play a significant role in angiogenesis was also found in previous studies to be associated with the risk of ONJ [11]. We also identified a novel gene associated with the risk of BRONJ that is involved in angiogenesis in patients of cancer BRONJ, *PLVAP*, which is the *VEGFA* downstream signaling target involved in the structure of the diaphragm and functions in vascular fenestrations [32]. Other genes identified in cancer group also have ONJ related functions: the *PZP* and *LTBP1* genes are involved in TGF-β signaling (which plays an important role in bone remodeling and tissue repair), the *HEBP1* gene is involved in heme pathophysiology, and the *ARID2* gene is involved in osteoblast differentiation. These findings suggest that exposure to high-dose BPs in patients with cancer with dysfunctional genes with various underlying pathophysiologies of ONJ increase the risk of BRONJ occurrence. On the other hand, in the osteoporosis BRONJ with a relatively long-term exposure to BPs (42.7 ± 46.3 months), there were no more candidate genes to explain the pathophysiology besides *VEGFA*.

Previous studies investigated to identify the contributing genetic profile of BRONJ development include two GWAS studies [10, 33], eleven candidate gene studies [11–15, 17, 34–38], and two WES studies [9, 18]. Most of these studies were case–control studies involving less than one hundred single-race patients for BRONJ in cancer patients. The candidate genes and SNPs identified through these studies varied and rarely replicated in another. The pathogenesis of BRONJ is not clearly defined, however, some hypothesis has been suggested [39, 40]. First, BPs strongly inhibits the activity of osteoclasts and induced apoptosis of osteoclasts. This reduces both bone absorption and formation. Second, BPs inhibit angiogenesis reducing blood vessel distribution in the bone along with inhibiting endothelial growth factor, which interferes with bone remodeling and wound healing in the jaw bone. Lastly, owing to the strong affinity of BPs to hydroxyapatite and long half-life leads to extreme suppression of bone turnover as well as wound healing. As the results of our study suggested as well, damaged genes involved in different but still diverse underlying mechanisms might contribute to the development of BRONJ with diverse mechanisms especially in patients with distinct underlying diseases with very different dosage and potency of BPs.

We used three analysis pipelines to identify candidate genes in order to minimize the false negative caused by various effects of causative genes and genetic variations contributing to BRONJ. The statistical technique using the traditional genetic model showed that the stop-gain mutation (rs117889746) in exon 15 of the *PZP* gene was significantly associated with the occurrence of BRONJ in 2 of the 13 cancer patients (1 homozygous and 1 heterozygous). These two patients developed ONJ after receiving Zoledronate injections for 10 months and 24 months after dental procedures such as extraction and implant removal. The *PZP* protein as a pan protease inhibitor is involved in the main mechanisms underlying the development of BRONJ: bone formation and inflammation. *PZP* protein is similar to α2-macroglobulin and has a high affinity with TGF-β1 and TGF-β2. Binding by *PZP* prevents TGFs from binding to cell-surface receptors, which in turn can eliminate TGF-β according to the morphological changes in *PZP*, and also act as a carrier [41]. TGF-β promotes tissue repair by enhancing the transcription of type I collagen, which is the main component of the extracellular matrix (ECM). Previous studies have shown that the expression of TGF-β is significantly reduced in specimens obtained from patients with nontraumatic osteonecrosis of the femoral head [42]. This is consistent with previous immunohistochemistry studies of the TGF-β1 signaling molecule in BRONJ patients showing significantly reduced TGF-β1 and Smad-2/3 in BRONJ patients compared to osteoradionecrosis patients [43]. Previous studies have shown that TGF-β promotes bone resorption of the mouse calvariae bone resorption at low doses and does not promote the resorption of the long bones at high doses [44]. Therefore, the results of our study suggest that the TGF-β signaling involved in ECM repair is related to the occurrence of BRONJ.

Our utilization of a gene-score analysis pipeline allowed us to identify more candidate genes than when using traditional genetic models. Excluding genes without known specific functions (*CDC27* and *TNRC18*), *ARID2*, *HEBP1*, *LTBP1*, and *PLVAP* were the only significant differences in the cancer group revealed by the gene-score methodology, while *DFFA*, *FAM193A*, and *VEGFA* were the only significant differences in the osteoporosis group. In particular, the *VEGFA* gene, which differed significantly in the osteoporosis BRONJ, is a member of previous well known risk gene families in ONJ [12], *VEGF* which is a growth factor that plays an important role in angiogenesis, vasculogenesis, and epithelial cell growth [42]. It has long been known that *VEFG* plays an important role in bone formation and repair [41], and there has also been a GWAS supporting the hypothesis that the impairment of angiogenesis in the tissue surrounding unnecrotized tissue would be involved in the development of BRONJ [11]. In addition, the *PLVAP* gene, which is involved in the structure of the diaphragm and vascular fenestrations identified in the cancer group, may be a downstream target of *VEGF* signaling, and is also an important factor in angiogenesis. Otherwise, the *LTBP1* gene that is related to osteoclast activity, which is involved in bone remodeling and the development of BRONJ, has been newly identified in this study. This gene has been shown to release the active form of TGF-β1 in the ECM [45], and it plays an important role in osteogenesis and bone resorption. Thus, dysfunction of the *LTBP1* gene might also be implicated in the development of BRONJ. The *HEBP1* gene identified in three BRONJ cancer patients is very interesting as well. This gene codes Heme Binding Protein 1 (HBP1), and heme is a complex of iron and tetrapyrrole protoporphyrin IX, which is in the prosthetic group of hemoproteins that play a key role in oxygen binding and the transportation of compounds such as hemoglobin and myoglobin [46]. An elevated concentration of free heme can induce pro-oxidant, proinflammatory, and cytotoxic effects that affect different cell types. Heme toxicity plays a major role in the pathogenesis of hemolytic disorders such as sickle-cell disease. Only a few studies have investigated the effects of dysfunction of the *HEBP1* gene on HBP production and metabolism, but heme toxicity and BRONJ present with very similar symptoms, and so further studies are needed into this association.

## Conclusions

We identified genes enriched significantly different between cancer and osteoporosis BRONJ group. Despite the small number of patients, the genes related to the pathophysiology in BRONJ occurrence were more enriched in the cancer group than osteoporosis. The limitations of our study are that we could not prove candidate genes and mutations derived from the study through additional testing besides the single ethnicity of participants (all East Asian). The results of this study need to be verified in future replication studies. It is well known that high doses of BPs increase the risk of BRONJ. Our results suggest that BRONJ may occur more easily in patients with impaired function of angiogenesis, osteoclast activity, and tissue repair with high dose BPs. If additional studies are conducted on a more sufficient patient population, the conclusions of this study should be supported.

## Supplementary information

**Additional file 1.** Suuplementary information.

## Data Availability

The datasets generated and/or analysed during the current study are not publicly available due to the consent limitation of the study participants but are available from the corresponding author on reasonable request.
