# Supplementary material for: Identifying genetic variants underlying medication-induced osteonecrosis of the jaw in cancer and osteoporosis: a case control study
Source: J Transl Med. 2019 Nov 20;17:381. doi: 10.1186/s12967-019-2129-3 (PMC6868688; doi:10.1186/s12967-019-2129-3)
Supplement: Supplementary file 1 — Additional file 1. Suuplementary information. [file 12967_2019_2129_MOESM1_ESM.docx]

**Additional Materials and Methods**

**Exome sequencing and variant calls**

Exome sequencing was performed using the Ion AmpliSeq™ Exome panel to screen coding sequence regions of the entire genome. This panel included the exome of 19,072 genes, and the size of the total targeted region was 57.7 Mb. The panel contained 293,903 primer pairs that were multiplexed into 12 pools to avoid primer-dimer formation and interference during PCR. The range of amplicons amplified by these oligo primer pairs ranged from 125 to 275 bp, and the rate of on-target coverage for this panel was 95.69%. PCR assays were performed directly to amplify 100 ng of genomic DNA samples extracted from peripheral blood cells so as to collect the target regions using the oligo primer pairs of the panel. The PCR conditions were as follows: 99°C for 2 min, followed by 10 cycles of 99°C for 15 sec, 60°C for 16 min, and 10°C for 1 min. After amplification, a library was constructed using the Ion Ampliseq Library Kit Plus as described in the manufacturer’s instructions (Thermo Scientific, Waltham, MA). Libraries were quantified using the Agilent 2100 Bioanalyzer (Agilent, Santa Clara, CA) and then diluted to ~10 pM. Subsequently, 50.0 μL of the barcoded libraries were combined into sets of two barcodes. The combined libraries were sequenced using the Ion Proton platform with PI Chip v3 following the manufacturer’s instructions (Thermo Scientific). Mapped reads to the human reference genome build (hg19) were generated by Torrent Suite Software v5.0.2 with germ-line and low-stringency settings. A mean depth of sequencing that ranged from 120× to 140× was obtained. Single-nucleotide polymorphism (SNP) variants and short insertions/deletions (INDELs) were identified using the Genome Analysis Toolkit 2.8-1 UnifiedGenotyper (McKenna et al. 2010). Raw reads were aligned to the reference human genome (hg19/GRCh37).

**Variant quality control and annotation**

After identifying variant calls, individual VCF files were merged using VCFtools ( Danecek et al. 2011), and quality-control (QC) filtering criteria were applied. All SNPs with a depth of coverage of <10, a mean mapping quality (MQ) of <40, and strand bias as estimated using a value from Fisher’s exact test (FS) of <60 were filtered out. INDELs with a mean MQ of <40 and FS <200 were filtered out. All of the QC filtered variants (SNPs and INDELs) were annotated using SnpEff ( Cingolani et al. 2012), including the following genomic effects: splice-site acceptors, splice-site donors, INDEL frameshifts, INDEL nonframeshifts, and nonsense (stop gain/loss), nonsynomymous, and synomymous variants. The population allele frequencies were annotated from the Exome Variant Server (Exome variant server) and 1000 Genomes Project (1000 Genomes Project Consortium). We also performed annotation according to in silico prediction algorithms, such as Sorting Intolerant From Tolerant (SIFT) ( NG et al. 2003), Polymorphism Phenotyping v2.2.2 (PolyPhen2) ( Kircher et al. 2014), Combined Annotation Dependent Depletion v1.3 (CADD) ( Adzhubei et al. 2010), and dbSNP 37 ( Sherry et al. 2001). All of the in silico prediction algorithms and population allele frequencies were annotated using SnpEff except for SIFT, which was downloaded from the SIFT Human database that supports GRCh37 Ensembl release 63 (the latest version) distributed by the J. Craig Venter Institute (<http://sift.jcvi.org/>).

**Study Design**

**
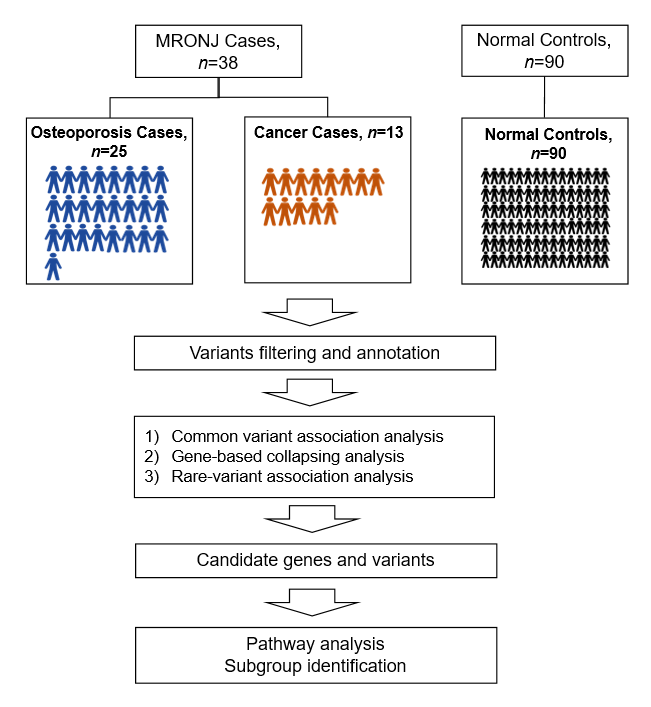
**

**Figure S1. Study design for MRONJ candidate gene and variant discovery analyses.**

**Additional Results**

**Identification of significantly altered genes**

Among the total of 38 cases, 188,111 SNPs and 532,725 INDELs were detected. Applying the QC filter criteria resulted in 159,236 SNPs and 461,658 INDELs remaining. To exclude likely false positives, we reviewed all significant variants identified in the three different analyses by manually inspecting all reads at all candidate loci in BAM files.

**Excluding genes in gene-score comparison analysis**

There were 16,010 genes in the MC and 16,281 genes in the MO that had at least 1 SIFT-score-annotated variant for each case. The genes that had a lower gene score in the cases than controls and had been corrected for multiple-tests bias comprised 284 genes for the MC versus controls and 645 genes for the MO versus controls with statistically significant differences. To eliminate hypervariable transcription-factor genes or proteins with under-defined functions, 10 ORF genes, 8 FAM genes, 13 olfactory genes, and 20 zinc-finger genes were excluded from the gene-score test results of the MC versus control group, and 19 ORF genes, 8 FAM genes, 14 olfactory genes, and 40 zinc-finger genes were excluded from the corresponding results for MO.

To identify contributing variants for cases with lower gene scores, we performed a Fisher’s exact test of the allele frequency of all variants with SIFT scores of <0.3 among the functionally damaging variants in the case versus control groups. Functionally damaging variants are defined in the Appendix Figure 1. At this step there were 10,087 and 12,952 variants for MC and MO, respectively, among which the number of intersection of variants having significantly different gene scores were 878 and 1,647, respectively. Then 161 variants in the cancer group and 444 variants in the osteoporosis group with higher allele frequency for cases than controls (*p*<0.05, odds ratio >1) were filtered in. To filter out population major variants and false-positive results, we excluded variants with lower allele frequencies in 90 healthy controls than in the ExAC Asian or the 1000 Genomes Project Asian population (12 variants in the cancer group and 15 variants in the osteoporosis group). After removing the variants with a zero allele count in the control group, six and five variants were identified and validated using IGV viewer for the MC and MO, respectively.


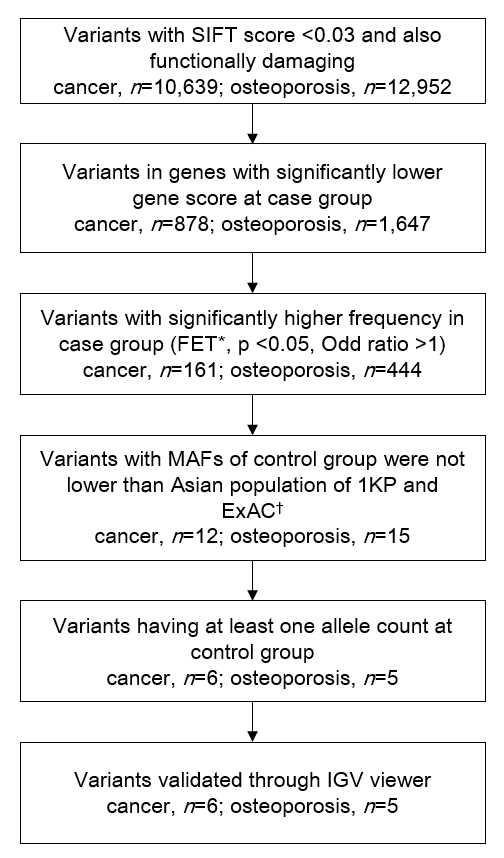


**Figure S2. Variant exclusion pipeline in gene-score comparison**

*FET, Fisher’s exact test, †ExAC, Exome Aggregation Consortium

**Table S1.** Results of the biological function clustering analysis of significant gene sets from gene-score comparisons for MRONJ osteoporosis cases versus controls.

| Group | Cluster no. (enrichment score) | Functional term | Category | No. of genes | Genes | *p* |
| --- | --- | --- | --- | --- | --- | --- |
| MO vs Control | 1 (1.38) | p130Cas linkage to MAPK signaling for integrins | R-HSA-372708 | 4 | VWF, PTK2, RAP1A, FN1 | 0.008 |
|  |  | MAP2K and MAPK activation | R-HSA-5674135 | 5 | CNKSR2, VWF, ARAF, RAP1A, FN1 | 0.027 |
|  | 2 (1.42) | EPHB-mediated forward signaling | R-HSA-3928662 | 7 | ACTR3, PTK2, ROCK1, LIMK2, WASL, YES1, EPHB1 | 0.001 |
|  |  | Ephrin receptor signaling pathway | GO:0048013 | 9 | ACTR3, PTK2, ANKS1A, ROCK1, EFNA4, WASL, YES1, MMP2, EPHB1 | 0.003 |
|  | 3 (1.27) | Rho-cell motility signaling pathway | H_rhoPathway | 5 | ACTR3, ARHGAP5, ROCK1, MYLK, ARHGEF11 | 0.031 |
|  | 4 (1.17) | Transcriptional activator activity, RNA polymerase II core promoter proximal region sequence-specific binding | GO:0001077 | 14 | EGR1, ESRRA, SSBP2, PTF1A, ARID3B, TEAD1, ELK1, NR4A1, TP73, MEF2D, FUBP3, POU2F3, HOXA7, NFATC2 | 0.017 |
|  | 5 (0.93) | Neural-crest-cell migration | GO:0001755 | 5 | SEMA6B, LAMA5, SEMA3G, SEMA3A, ALX1 | 0.037 |
|  | 6 (0.90) | Wnt signalosome | GO:1990909 | 3 | FZD1, CTNNB1, DVL1 | 0.035 |
|  | 7 (0.88) | Sema4D-induced cell migration and growth-cone collapse | R-HSA-416572 | 4 | ROCK1, LIMK2, RHOB, ARHGEF11 | 0.035 |
|  | 8 (0.69) | Protein sumoylation | GO:0016925 | 8 | BMI1, SENP2, POM121, SEH1L, NUP88, MDC1, SP3, STAG1 | 0.047 |
|  | 9 (0.68) | Rab GTPase binding | GO:0017137 | 9 | TBC1D25, TBC1D16, GDI1, EVI5, ATP6AP1, VPS52, ODF2, SYTL1, TBC1D8B | 0.036 |
|  | 10 (0.68) | Lysosome | HSA04142 | 9 | TCIRG1, AGA, LAMP1, NPC1, ATP6AP1, PPT2, ATP6V0A1, ACP2, GGA2 | 0.027 |
|  | 11 (0.42) | Negative regulation of canonical Wnt signaling pathway | GO:0090090 | 13 | EGR1, HECW1, IGFBP6, FZD1, STK3, DVL1, PSMC5, GPC3, DACT3, PFDN5, PSMB2, RAPGEF1, PSMD9 | 0.002 |

**Table S2.** Results of the biological function clustering analysis of significant gene sets from gene-score comparisons for MC versus controls

| Group | Cluster no. (enrichment score) | Functional term | Category | No. of genes | Genes | *p* |
| --- | --- | --- | --- | --- | --- | --- |
| MC vs Control | 1 (1.62) | Transcription-factor complex | GO:0005667 | 10 | GATA6, RCOR2, MTA2, YY1, ONECUT3, PTF1A, NR4A1, TEAD2, CTNNB1, ALX1 | <0.001 |
|  |  | Positive regulation of transcription from RNA polymerase II promoter | GO:0045944 | 21 | PHOX2A, ESRRA, SOX11, ONECUT3, YY1, MTA2, PTF1A, TGFB3, NR4A1, TEAD2, SOX9, CSRP3, CBFB, CTNNB1, MEF2D, TET3, GATA6, BRD4, YES1, AKIRIN2, ALX1 | 0.007 |
|  | 2 (1.56) | Spliceosome | HSA03040 | 9 | PRPF8, USP39, CDC40, HSPA6, SYF2, SNRPC, RBMXL3, SF3B4, SF3B2 | <0.001 |
|  |  | mRNA splicing, via spliceosome | GO:0000398 | 8 | FRG1, PRPF8, USP39, CDC40, SYF2, SNRPC, SF3B4, SF3B2 | 0.013 |
|  |  | Catalytic step 2 spliceosome | GO:0071013 | 5 | FRG1, PRPF8, CDC40, SYF2, SF3B2 | 0.019 |
|  |  | Spliceosomal complex | GO:0005681 | 5 | PRPF8, USP39, CDC40, SF3B4, SF3B2 | 0.021 |
|  |  | Pre-mRNA splicing, U2-dependent splicing: mRNA splicing | R-HSA-72163 | 7 | PRPF8, USP39, CDC40, SYF2, SNRPC, SF3B4, SF3B2 | 0.023 |
|  |  | RNA splicing | GO:0008380 | 6 | PRPF8, USP39, CDC40, SREK1IP1, SF3B4, SF3B2 | 0.039 |
|  | 3 (1.24) | DAP12 signaling | R-HSA-2424491 | 4 | LAT, KLRC2, PLCG1, GRAP2 | 0.006 |
|  |  | Generation of second-messenger molecules | R-HSA-202433 | 4 | LAT, PLCG1, HLA-DRB5, GRAP2 | 0.010 |
|  |  | T-cell-receptor signaling pathway | GO:0050852 | 6 | LAT, PLCG1, PSMB2, HLA-DRB5, GRAP2, PSMD9 | 0.026 |
|  | 4 (0.89) | Natural-killer-cell-mediated cytotoxicity | hsa04650 | 6 | LAT, TNFRSF10B, PLCG1, PRKCG, KIR2DS4, SHC2 | 0.016 |
|  | 5 (0.77) | Cell–cell junction | GO:0005911 | 6 | ACTR3, LAT, PLCG1, PRKCG, AQP7, CTNNB1 | 0.043 |

**Table S3. Genes using in biological clustering from the SKAT-O test in osteoporosis group**

| Hugo | m_pvalue | o_pvalue | b_pvalue | fdr_m_pvalue | fdr_o_pvalue | fdr_b_pvalue |
| --- | --- | --- | --- | --- | --- | --- |
| ABCA13 | 0.500919 | 0.00298 | 0.500007 | 0.543652 | 0.052786 | 0.558657 |
| ADGRF2 | 0.013178 | 0.00746 | 0.500004 | 0.060313 | 0.052786 | 0.558657 |
| ANXA1 | 0.010519 | 0.012479 | 0.500213 | 0.060005 | 0.059399 | 0.558657 |
| ARHGEF38 | 0.047922 | 0.033537 | 0.500013 | 0.100006 | 0.08917 | 0.558657 |
| ASPN | 0.002961 | 0.005323 | 0.500004 | 0.053533 | 0.052786 | 0.558657 |
| ATP6V1G1 | 0.501336 | 0.00754 | 0.500012 | 0.543652 | 0.052786 | 0.558657 |
| CASQ1 | 0.007911 | 0.005714 | 0.500081 | 0.060005 | 0.052786 | 0.558657 |
| CD300C | 0.039462 | 0.035711 | 0.500029 | 0.095252 | 0.08917 | 0.558657 |
| CDC20B | 0.007623 | 0.005455 | 0.500023 | 0.060005 | 0.052786 | 0.558657 |
| CEP162 | 0.050423 | 0.048698 | 0.500317 | 0.100006 | 0.098206 | 0.558657 |
| CLEC5A | 0.038169 | 0.038169 | 0.500086 | 0.095252 | 0.08917 | 0.558657 |
| CPNE1 | 0.031359 | 0.013183 | 0.500004 | 0.095252 | 0.06034 | 0.558657 |
| CT45A10 | 0.501707 | 0.003032 | 0.500016 | 0.543652 | 0.052786 | 0.558657 |
| DIAPH3 | 0.033761 | 0.041267 | 0.500028 | 0.095252 | 0.092656 | 0.558657 |
| EFCAB13 | 0.047865 | 0.049515 | 0.500011 | 0.100006 | 0.098206 | 0.558657 |
| ENDOU | 0.034011 | 0.027867 | 0.500052 | 0.095252 | 0.08917 | 0.558657 |
| FGGY | 0.008878 | 0.009352 | 0.102207 | 0.060005 | 0.05501 | 0.558657 |
| GPAA1 | 0.010519 | 0.008461 | 0.500213 | 0.060005 | 0.052992 | 0.558657 |
| GRK4 | 0.00737 | 0.00737 | 0.127039 | 0.060005 | 0.052786 | 0.558657 |
| GSTM3 | 0.045089 | 0.027092 | 0.500305 | 0.100006 | 0.08917 | 0.558657 |
| HIST1H2AH | 0.01553 | 0.030124 | 0.500493 | 0.068446 | 0.08917 | 0.558657 |
| HRNR | 0.501368 | 0.003009 | 0.500012 | 0.543652 | 0.052786 | 0.558657 |
| IL20 | 0.013147 | 0.002942 | 0.500002 | 0.060313 | 0.052786 | 0.558657 |
| KIF17 | 0.041813 | 0.047593 | 0.5013 | 0.095686 | 0.097647 | 0.558657 |
| KLK10 | 0.028191 | 0.032587 | 0.500564 | 0.095252 | 0.08917 | 0.558657 |
| KRBA2 | 0.033276 | 0.035199 | 0.500002 | 0.095252 | 0.08917 | 0.558657 |
| MFSD3 | 0.031878 | 0.040231 | 0.500916 | 0.095252 | 0.092067 | 0.558657 |
| MGST2 | 0.011093 | 0.01671 | 0.500347 | 0.060005 | 0.073649 | 0.558657 |
| MMP8 | 0.033491 | 0.027402 | 0.500011 | 0.095252 | 0.08917 | 0.558657 |
| MRPL9 | 0.010409 | 0.010409 | 0.500187 | 0.060005 | 0.05501 | 0.558657 |
| MYOC | 0.007057 | 0.006805 | 0.100915 | 0.060005 | 0.052786 | 0.558657 |
| NOX3 | 0.499682 | 0.005254 | 0.5 | 0.543652 | 0.052786 | 0.558657 |
| OR2AG1 | 0.047865 | 0.027402 | 0.500011 | 0.100006 | 0.08917 | 0.558657 |
| OR4K13 | 0.04939 | 0.046066 | 0.500006 | 0.100006 | 0.096173 | 0.558657 |
| OR5T1 | 0.013032 | 0.011094 | 0.500347 | 0.060313 | 0.05501 | 0.558657 |
| PCTP | 0.002924 | 0.005273 | 0.500001 | 0.053533 | 0.052786 | 0.558657 |
| PDZD3 | 0.031878 | 0.038216 | 0.500916 | 0.095252 | 0.08917 | 0.558657 |
| PMF1-BGLAP | 0.028191 | 0.028191 | 0.500564 | 0.095252 | 0.08917 | 0.558657 |
| PPM1F | 0.040022 | 0.020826 | 0.500866 | 0.095252 | 0.08851 | 0.558657 |
| PRR5-ARHGAP8 | 0.009507 | 0.005349 | 0.500007 | 0.060005 | 0.052786 | 0.558657 |
| PRSS42 | 0.010409 | 0.010409 | 0.500187 | 0.060005 | 0.05501 | 0.558657 |
| RNF175 | 0.049704 | 0.04603 | 0.500916 | 0.100006 | 0.096173 | 0.558657 |
| SDR39U1 | 0.03459 | 0.03142 | 0.105571 | 0.095252 | 0.08917 | 0.558657 |
| SERHL2 | 0.031878 | 0.036154 | 0.500916 | 0.095252 | 0.08917 | 0.558657 |
| SH2D4A | 0.033491 | 0.027402 | 0.500011 | 0.095252 | 0.08917 | 0.558657 |
| SIGLEC1 | 0.036998 | 0.042401 | 0.500001 | 0.095252 | 0.093438 | 0.558657 |
| SMC5 | 0.029062 | 0.035569 | 0.246642 | 0.095252 | 0.08917 | 0.558657 |
| SPHK1 | 0.039953 | 0.031595 | 0.50085 | 0.095252 | 0.08917 | 0.558657 |
| SPTB | 0.036998 | 0.031233 | 0.500001 | 0.095252 | 0.08917 | 0.558657 |
| STAB2 | 0.011093 | 0.011093 | 0.500347 | 0.060005 | 0.05501 | 0.558657 |
| STARD5 | 0.041489 | 0.037807 | 0.500046 | 0.095686 | 0.08917 | 0.558657 |
| STPG1 | 0.007541 | 0.007541 | 0.500012 | 0.060005 | 0.052786 | 0.558657 |
| TFDP3 | 0.499682 | 0.00291 | 0.5 | 0.543652 | 0.052786 | 0.558657 |
| TLR2 | 0.047865 | 0.035422 | 0.500011 | 0.100006 | 0.08917 | 0.558657 |
| TMPRSS7 | 0.500088 | 0.002932 | 0.500001 | 0.543652 | 0.052786 | 0.558657 |
| TRIM65 | 0.029462 | 0.044448 | 0.50001 | 0.095252 | 0.096169 | 0.558657 |
| ZNF165 | 0.018486 | 0.027307 | 0.500353 | 0.075855 | 0.08917 | 0.558657 |
| ZNF175 | 0.01828 | 0.027085 | 0.500303 | 0.075855 | 0.08917 | 0.558657 |
| ZNF30 | 0.012413 | 0.008393 | 0.500197 | 0.060313 | 0.052992 | 0.558657 |
| ZNF350 | 0.035808 | 0.031527 | 0.500833 | 0.095252 | 0.08917 | 0.558657 |

**References**

Adzhubei IA, Schmidt S, Peshkin L, Ramensky VE, Gerasimova A, Bork P, Kondrashov AS, Sunyaev SR. 2010. A method and server for predicting damaging missense mutations. Nat Methods. (4):248–9.

Cingolani P, Platts A, Wang LL, Coon M, Nguyen T, Wang L, Land SJ, Lu X, Ruden DM. 2012. A program for annotating and predicting the effects of single nucleotide polymorphisms, SnpEff: SNPs in the genome of Drosophila melanogaster strain w ^1118^ ; iso-2; iso-3. Fly (Austin). 6(2):80–92.

Danecek P, Auton A, Abecasis G, Albers CA, Banks E, DePristo MA. Handsaker RE, Lunter G, Marth GT, Sherry ST, McVean G, Durbin R; 1000 Genomes Project. 2011. Analysis Group.The variant call format and VCFtools. Bioinformatics. (15):2156–8.

Exome Variant Server, NHLBI GO Exome Sequencing Project (ESP), Seattle, WA (URL: http://evs.gs.washington.edu/EVS/) [Jan 2018 accessed].

Kircher M, Witten DM, Jain P, O’Roak BJ, Cooper GM, Shendure J. 2014. A general framework for estimating the relative pathogenicity of human genetic variants. Nat Genet. 46(3):310–5.

McKenna, Aaron, et al. 2010. The Genome Analysis Toolkit: a MapReduce framework for analyzing next-generation DNA sequencing data. Genome Res (9): 1297-1303. Ng, Pauline C., and Steven Henikoff. 2003. SIFT: Predicting amino acid changes that affect protein function." Nucleic Acids Res (13): 3812-3814

Sherry ST, Ward M-H, Kholodov M, Baker J, Phan L, Smigielski EM, Sirotkin K.2001. dbSNP: the NCBI database of genetic variation. Nuc Acids Res. 29(1):308–11.

1000 Genomes Project Consortium, A. Auton, and L. D. Brooks. 2015. A global reference for human genetic variation." Nature (7571): 68-74.
